# Editorial: Innovative Biologics and Drugs to Target Renal Inflammation

**DOI:** 10.3389/fphar.2020.00038

**Published:** 2020-02-06

**Authors:** Matthew D. Griffin, Sundararaman Swaminathan

**Affiliations:** ^1^Regenerative Medicine Institute (REMEDI) at CÚRAM SFI Research Centre, National University of Ireland Galway, Galway, Ireland; ^2^Division of Nephrology, University of Virginia Health System, Charlottesville, VA, United States

**Keywords:** Chronic Kidney Disease, Acute Kidney Injury, Inflammation, Therapeutic Targets, Diabetic Nephropathy, Sepsis, Interstitial Fibrosis, Biologic Agents

The prevalences of acute kidney injury (AKI), chronic kidney disease (CKD), and end-stage renal disease (ESRD) are increasing globally and are associated with an escalating socioeconomic burden (Jha et al., 2013; Wang et al., 2016; Hoste et al., 2018). It is now well recognized that acute and chronic loss of kidney function lead to dramatically increased risk for cardiovascular events and death and that CKD is associated with significantly poorer health-related quality of life (Wang et al., 2016; Lv and Zhang, 2019; See et al., 2019). These fundamental insights into the worldwide trends and consequences of kidney disease highlight the importance of research focussed on developing new strategies for the prevention and treatment of AKI and CKD.

One important pathophysiological process that links virtually all forms of kidney disease and their cardiovascular and other complications is inflammation (Swaminathan and Shah, 2011; Kurts et al., 2013; Rabb et al., 2016; Sarnak et al., 2019). It is now well recognized that production of inflammatory mediators is directly triggered by programmed cell death pathways, metabolic dysfunction and endoplasmic reticulum stress within renal parenchymal cells and results in exacerbation of tissue injury during AKI and CKD (Mulay et al., 2016; Sarnak et al., 2019). Furthermore, dysregulated immune cells contribute to progressive fibrosis of the kidney as well as to acceleration of vascular injury in CKD/ESRD (Kurts et al., 2013; Mulay et al., 2016; Rabb et al., 2016; Sarnak et al., 2019). Over the past two decades, advancing knowledge of intra-renal inflammatory pathways and of resident and recruited immune cell populations within the kidneys has unearthed new opportunities for the development of mechanistically informed, anti-inflammatory therapies for kidney disease (Kurts et al., 2013). More recently, evidence has emerged from a small number of clinical trials, that such strategies can be effective in slowing the progression of renal functional loss (Perez-Gomez et al., 2016; Menne et al., 2017; Nowak et al., 2017). The goal of this Research Topic was to highlight recent basic, pre-clinical, and clinical progress and opportunities related to the targeting of renal inflammation using drugs and biologic agents by publishing relevant full-length and short original research communications and review articles. In the remaining sections of this editorial, we briefly describe and discuss the implications of the eight excellent contributions made to the Research Topic by groups of investigators from Asia, Europe, and North and South America.

We first wish to highlight the comprehensive review article contributed by Andrade-Oliveria et al. In the first part of their review, the authors provide an up-to-date summary of the “classical” innate and adaptive immune cell types, pattern recognition receptors, and signalling pathways that have been shown to mediate positive and negative effects on renal parenchymal injury and distant organ responses in models of AKI, CKD, and the transition between the two. Next, they present an excellent synopsis of the compelling evidence for important links between the gut microbiome, its metabolic products and inflammatory activities associated with kidney disease. Current progress toward the application of therapeutic strategies for altering the course of CKD or its complications by manipulating the gut microbiome and/or gut microbe-derived metabolites is also described. Finally, the authors emphasize the less well recognized anti-inflammatory effects of established drug classes such as renin angiotensin system (RAS) blocking agents and xanthine oxidase inhibitors. Overall, this review represents a very readable overview of the complexities of renal inflammation and the diversity of potential strategies for modulating it.

Three original research articles from this collection describe novel experimental strategies for ameliorating the harmful effects of inflammation in the setting of diabetes and diabetic nephropathy (DN) – the most common cause for CKD/ESRD worldwide (Jha et al., 2013). Based on evidence from a range of other tissues and disease settings (Besnard et al., 2013), Sun et al. investigated the potential role of the pro-inflammatory chemokine CXCL6 (a ligand for CXCR1 and CXCR2) in promoting renal interstitial fibrosis in DN. In their study, expression profiles of human kidney biopsy and plasma samples and from a high fat/high sugar diet + streptozotocin (STZ)-induced rat model of diabetes convincingly demonstrate that CXCL6 and related inflammation and fibrosis markers are increased in kidneys and circulation in the setting of DN with interstitial fibrosis. To better explore the underlying mechanisms, the authors then performed a series of experiments in a culture system using the NRK-49F rat kidney fibroblast cell line. These *in vitro* results confirmed that fibroblast expression of CXCL6 and CXCR1 was induced in dose-dependent fashion by exposure to high glucose concentration. More strikingly, high glucose-induced fibroblast proliferation and production of inflammatory and pro-fibrotic mediators were increased and decreased respectively by CXCL6 over-expression and suppression through a JAK/STAT-dependent mechanism. Thus, the study provides a novel evidence base for further pre-clinical and, potentially, clinical studies aimed at blocking CXCL6 during the early or later stages of diabetic kidney disease (DKD). Using a mouse STZ-induced model of diabetes, Yang et al. investigated the reno-protective effects of the anti-inflammatory plant-derived flavonoid myricetin during the onset of DN. Importantly, by comparing results for diabetic wild-type mice and mice in which the anti-oxidative transcription factor nuclear factor (erythroid derived 2)-like 2 (Nrf2) was knocked down, these authors tested the hypothesis that myricetin ameliorates DN severity through a Nrf2-mediated mechanism as had been postulated by others (Semwal et al., 2016). Diabetic and non-diabetic mice were treated orally twice daily for 6 months. Impressively, the myricetin-treated animals had substantial reductions in glomerular and interstitial pathological changes along with reduced expression of inflammatory, oxidative, and fibrotic mediators. While myricetin administration was also associated with increased nuclear translocation of Nrf2, its reno-protective effect was only partially reduced by Nrf2 knockdown. As a naturally-occurring agent, myricetin may be quite readily amenable to clinical testing for benefit to prevent or slow the progression on DKD. Taking a more targeted approach, Sabapathy et al. evaluated an innovative recombinant biologic for increasing regulatory T-cells (T-reg) in the setting of obesity-associated type 2 diabetes and DN in the *Ob* mouse strain. In this study, the authors demonstrate that a 5-day course of the chimeric cytokine IL233, which combines the T-reg-activating effects of interleukin (IL)-2 and IL-33 (Stremska et al., 2017), resulted in improved glycemic control, decreased weight gain and visceral fat accumulation, reduced albuminuria, and reduced intra-renal inflammation 8-13 weeks later compared to saline-treated *Ob* mice. Importantly, in addition to promoting a prolonged increase in T-regs at multiple tissue sites, IL233 was also associated with increased proportions of other potentially anti-inflammatory immune cells including T-helper type 2 (Th2) cells, type 2 innate lymphocytic cells, and alternatively-activated macrophages. This study provides an exciting example of the potential for novel immunotherapeutic strategies to be used to prevent DKD by modulating the chronic, systemic micro-inflammation associated with type 2 DM and obesity.

Two other original articles published within the Research Topic address the potential to slow the progression of CKD by manipulating recently-identified molecular mechanisms of pro-fibrotic renal inflammation. Orejudo et al. investigated the influence of the cytokine IL-17A, which may be produced within the kidneys by a pro-inflammatory CD4^+^ T-cell subtype, Th17 cells, as well as by innate immune cells including γδ T-cells and neutrophils (Cortvrindt et al., 2017) on hypertension and its associated renal injury. Among the findings reported by these authors were that 14-day infusion of IL-17A was associated with raised BP and increased infiltration of multiple immune cell types into the kidneys of mice, that IL-17A blockade with a neutralizing antibody reduced renal inflammation and fibrosis in mice infused with angiotensin II and that hypertension/hypertensive nephrosclerosis in rats and humans was associated with the presence of IL-17A^+^ cells within the interstitial compartment. Although more definitive *in vivo* evidence will be needed to determine whether blocking IL-17A or its downstream effects has the potential to slow the progression of hypertensive kidney disease, the clinical introduction of anti-IL17A monoclonal antibodies for immune-mediated inflammatory diseases (Balato et al., 2017) provides an interesting backdrop for this study. In the brief report of Li et al., the effect of supplementing klotho, a reno-protective protein that is typically down-regulated in the kidney in CKD, on renal fibrosis following unilateral ureteral obstruction was investigated in mice. In keeping with previously published work from the authors and others (Doi and Masaki, 2017), alternate day administration of recombinant klotho was associated with reduced renal fibrosis and transforming growth factor β1/Smad2 signalling compared to saline administration. More specifically, co-staining of kidney for endothelial and fibroblast markers suggested that klotho replenishment resulted in partial prevention of endothelial to mesenchymal transformation (EndoMT). Taken in the context of a large body of literature supporting a direct role of klotho downregulation in promoting chronic progression of renal fibrosis in a variety of disease settings (Doi and Masaki, 2017; Zou et al., 2018), this study provides further evidence for the potential to treat CKD by restoring or replacing this tissue-preserving protein or its downstream effects.

Finally, two articles in the Research Topic focus on AKI in the setting of sepsis—a clinical challenge for which novel therapeutic strategies are desperately needed to reduce the associated high rates of mortality and long-term morbidity (Peerapornratana et al., 2019). In an extensive original study from one of our own research groups, Scindia et al. utilized the mouse lipopolysaccharide (LPS) and cecal ligation and puncture (CLP) models of sepsis as well as mechanistic experiments in cultured cells, to reveal the potential for exogenous supplementation of the iron-sequestering protein hepcidin to prevent or treat sepsis-associated AKI (SA-AKI). Pre-treatment of mice 24 h prior to LPS administration resulted in potent reno-protection that was primarily mediated by an inhibitory effect on systemic inflammation. Additional experiments implicated a specific requirement for H-ferritin expressed by splenic macrophages in this anti-inflammatory mechanism. Importantly, the anti-inflammatory and reno-protective effects of hepcidin administration were replicated in the more clinically relevant CLP model of polymicrobial sepsis in experiments in which it was administered at two time-points prior to or after sepsis induction. This study contributes to a burgeoning body of evidence that iron metabolism and compartmentalisation is a key, targetable regulator of oxidative stress, cell death and inflammation in AKI and other kidney disease settings (Swaminathan, 2018). In their review article for this Research Topic, Hümmeke-Oppers et al. expertly summarize the pre-clinical and clinical evidence for a beneficial effect of another modulator of inflammation, the endogenous “detoxifying” enzyme alkaline phosphatase (AP), in SA-AKI. In the initial sections of the review, putative mechanisms of action of AP to dampen inflammation during microbial sepsis, including dephosphorylation of the inflammatory triggers LPS and extracellular ATP, are described along with animal model reports in support of a therapeutic effect. Subsequently, the authors summarize the results of phase 1 and phase 2 clinical trials of bovine intestinal AP and recombinant human AP that have provided strong evidence of safety and preliminary evidence of efficacy of this therapy to increase renal functional recovery and reduce mortality associated with SA-AKI. They speculate that prolonged benefits of AP administration during the early phase of SA-AKI may represent a modulatory effect on inflammation-associated renal repair mechanisms. Given that the original report of LPS detoxification by AP was published in 1997 (Poelstra et al., 1997), this incisive review from a group that has led the way in delivering key outcome data for AP-treated patients with SA-AKI, also illustrates the significant time-lines required to translate novel therapeutic strategies for acute and chronic kidney diseases to clinical practice.

## Conclusions

To conclude, the collection of articles contributed to this Research Topic illustrates the growing emphasis on inflammatory pathways and mediators as targetable elements of the complex pathophysiology of AKI and CKD. It is noteworthy that the research described in these articles spans basic studies that identify putative novel inflammatory mediators which may one day prove to be of clinical relevance to pre-clinical and clinical evidence of therapeutic value of targeting known mediators of one or more forms of kidney disease. These articles also serve to highlight that matching of novel fundamental insights into the mechanisms of renal inflammation and repair with technological advances in the design and delivery of targeted therapies or better defining the mechanisms of action of agents with known disease-modulating effects offers substantial hope for an accelerated pipeline of new treatments to prevent and slow the progression of kidney disease and its complications in the decades ahead.

## Author Contributions

MG contributed to the concept, design and drafting of the manuscript and its approval for publication. He agrees to be accountable for all aspects of the work in ensuring that questions related to the accuracy or integrity of any part of the work are appropriately investigated and resolved. SS contributed to the concept, design and critical revision of the manuscript and its approval for publication. He agrees to be accountable for all aspects of the work in ensuring that questions related to the accuracy or integrity of any part of the work are appropriately investigated and resolved.

## Conflict of Interest

The authors declare that the research was conducted in the absence of any commercial or financial relationships that could be construed as a potential conflict of interest.
